# When Bones Tell a Story: Diaphyseal Femoral Fracture in a Child With Pycnodysostosis

**DOI:** 10.7759/cureus.93781

**Published:** 2025-10-03

**Authors:** Kamal El Ghazy, Badr Rouijel, Mohammed Eljadid, Zineb Benmassaoud, Hind Cherrabi, Mohamed Amine Oukhouya

**Affiliations:** 1 Pediatric Surgery Department, Souss Massa University Hospital Center, Faculty of Medicine and Pharmacy of Agadir, Ibn Zohr University, Agadir, MAR; 2 Pediatric Surgery Department, Regional Hospital Center of Hassan II, Agadir, MAR

**Keywords:** cathepsin k, pediatric surgery, plate, pycnodysostosis, sclerotic bone

## Abstract

Pycnodysostosis (PYCD) is a rare genetic disorder marked by generalized bone sclerosis and a high risk of fractures. On average, children with PYCD experience approximately 0.2 fractures per year, often complicated by delayed healing and poor bone remodeling. Surgical treatment of long bone fractures in patients with PYCD is scarcely documented in the literature, and orthopedic surgeons play a crucial role in managing the affected children. In this article, we present the case of an 11-year-old boy diagnosed with PYCD who presented to the pediatric surgery department for the management of a femoral diaphysis fracture, and we discuss the therapeutic difficulties.

## Introduction

Pycnodysostosis (PYCD) is an uncommon hereditary skeletal disorder inherited in an autosomal recessive pattern, caused by mutations that result in a deficiency of the enzyme cathepsin K (CTSK) [1]. This enzymatic abnormality disrupts normal bone remodeling processes, leading to bones that are excessively dense yet fragile, making them susceptible to recurrent fractures. Individuals with PYCD typically exhibit distinct physical characteristics such as short stature, craniofacial abnormalities including frontal bossing, a prominent nose, delayed cranial suture closure, and dental irregularities. The narrow medullary canals seen in affected patients often complicate surgical management. Despite the high bone density, the brittleness of the bones presents significant challenges in fracture treatment, although healing potential remains considerable [2].

We present the case of an 11-year-old boy who was admitted to the pediatric surgery department at Souss Massa University Hospital Center for management of a pathological fracture of the right femoral diaphysis attributed to PYCD.

## Case presentation

The patient is an 11-year-old boy, born to a consanguineous marriage, with a prior history of a right leg fracture managed orthopedically with a satisfactory outcome. He presented following a minor trauma to the right thigh, causing localized pain and complete loss of function of the right lower limb.

**Figure 1:**
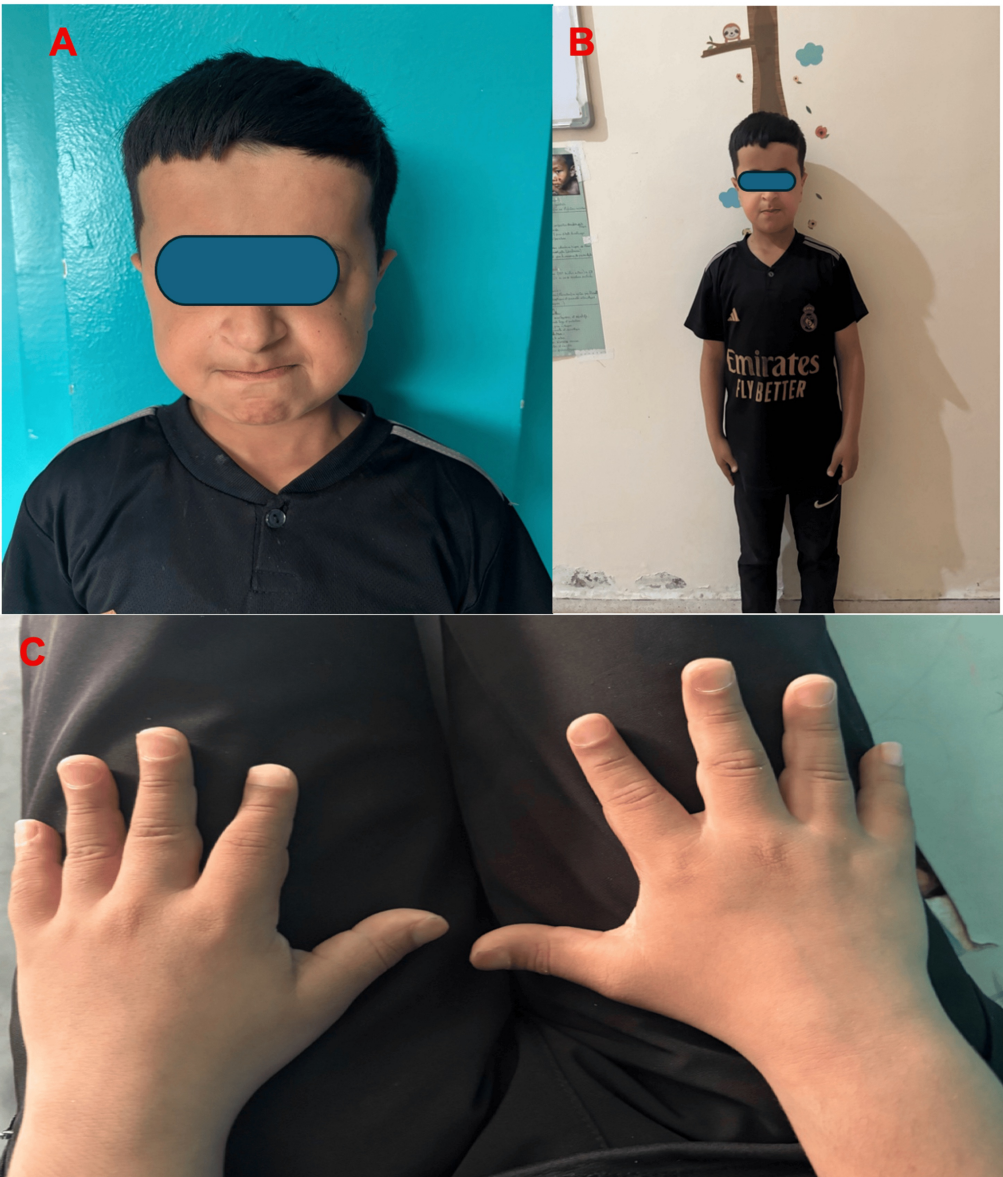
(A) Characteristic facial dysmorphism in pycnodysostosis, (B) statural retardation (-2DS), and (C) brachydactyly of both hands

On clinical examination, several characteristic features of PYCD were noted, including a dysmorphic facial appearance with frontal bossing and a prominent nose (Figure 1A), short stature (Figure 1B), and brachydactyly affecting both hands (Figure 1C).

**Figure 2:**
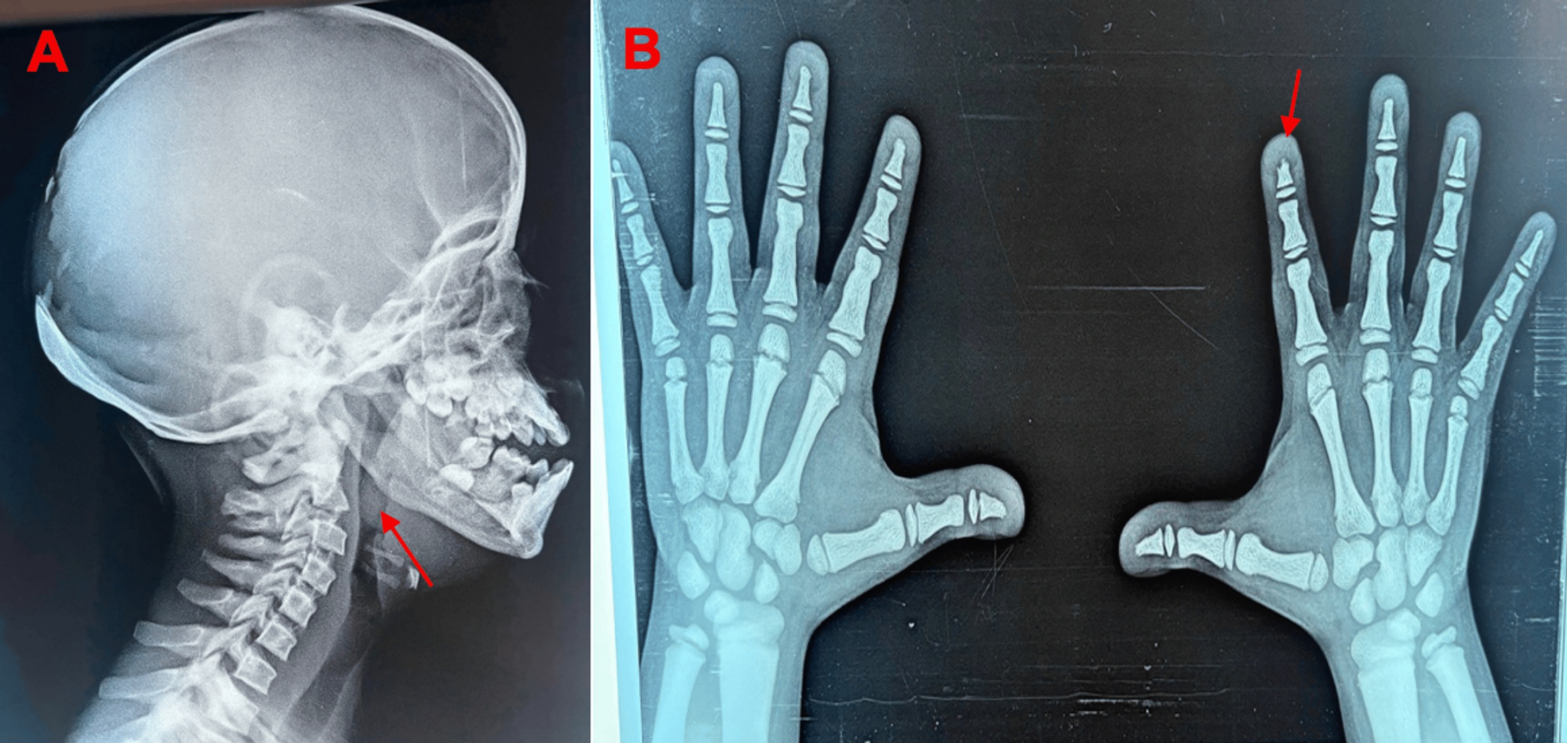
(A) Red arrow showing the obtuse angle of the mandible and (B) bilateral hand X-ray showing a short distal phalanx

Radiological assessment included evaluation of the craniofacial skeleton, revealing an obtuse-angled mandible (Figure 2A), and identification of shortened distal phalanges in several digits (Figure 2B), which are typical radiological findings in this condition.

**Figure 3:**
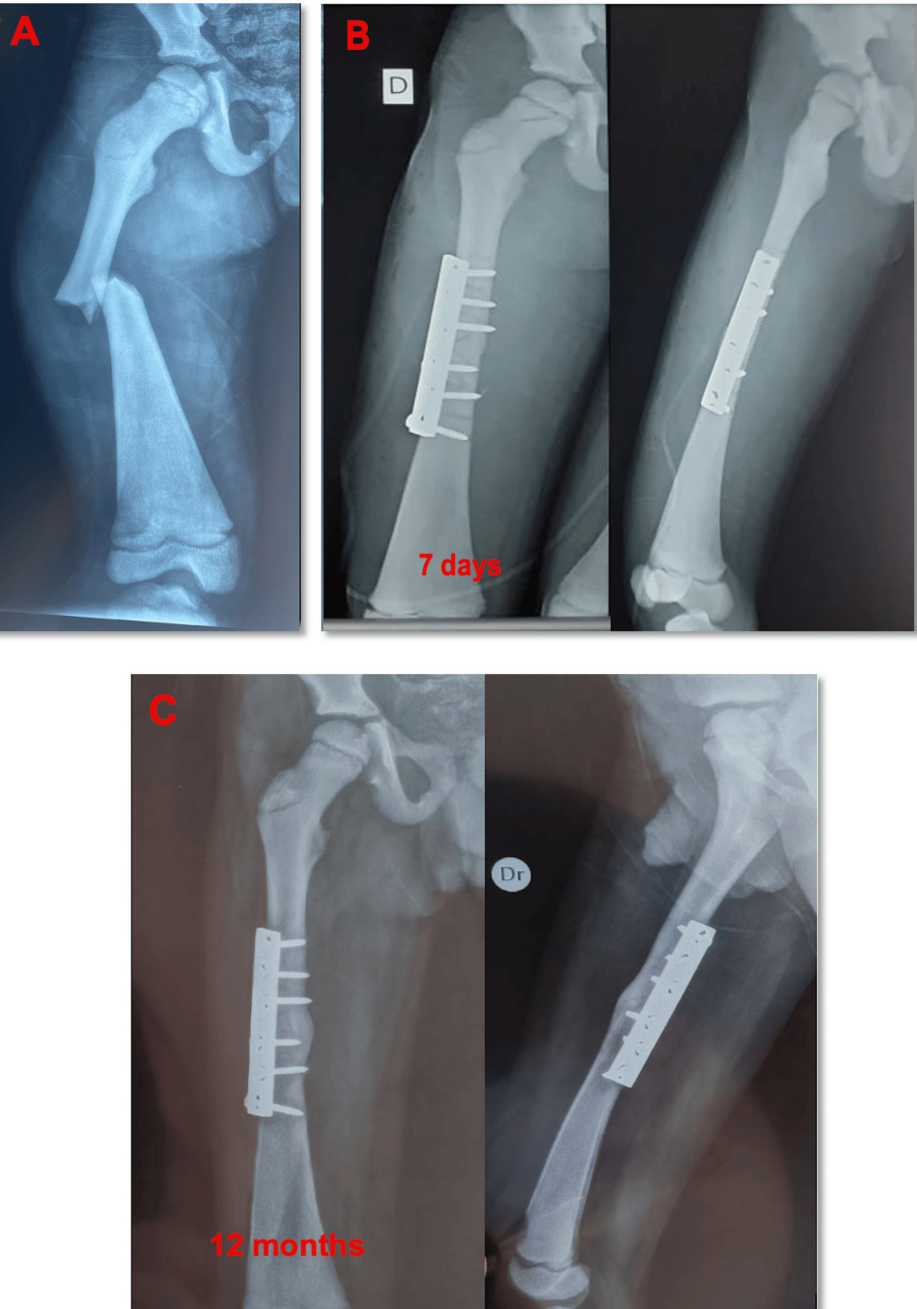
(A) A displaced transverse fracture of the right femoral diaphysis with a narrow medullary canal, (B) postoperative control radiographs after seven days, and (C) control radiographs after 12 months

Standard radiographs of the femur showed a transverse fracture at the mid-diaphysis (Figure 3A). The affected bone demonstrated abnormally increased density, consistent with the osteosclerotic nature of PYCD, and the medullary canal appeared markedly narrowed, a factor that complicates intramedullary fixation.

Given these anatomical challenges, an initial attempt was made to stabilize the fracture using elastic intramedullary nailing to provide minimally invasive fixation suitable for pediatric patients. However, the extremely sclerotic bone and the restricted medullary canal diameter made multiple attempts difficult. Consequently, the surgical plan was modified to proceed with open reduction and internal fixation using a locking compression plate, which allowed for stable fixation without reliance on the intramedullary canal.

The postoperative course was uneventful, with early recovery and no immediate complications. Follow-up radiographs at seven days showed appropriate alignment and hardware positioning (Figure 3B). Further radiological evaluation at 12 months post-surgery demonstrated satisfactory fracture consolidation and bone healing (Figure 3C), consistent with favorable surgical outcomes reported in patients with PYCD when rigid fixation methods are employed.

## Discussion

The estimated prevalence of PYCD is one in 1.7 million [3]. Initially characterized in 1962 by Maroteaux and Lamy, it is a kind of dwarfism accompanied by craniofacial anomalies such as cleidocranial dysostosis. Other authors have referred to it as the Toulouse-Lautrec syndrome, identifying the French painter Henri de Toulouse-Lautrec as an affected individual [4]. This is an autosomal recessive condition caused by a mutation in the gene that encodes CTSK.

Cathepsin K is an important lysosomal cysteine protease found in the bone matrix, playing a crucial role in bone resorption by osteoclasts. Mutations in the CTSK gene cause a deficiency of this enzyme, resulting in osteoclast dysfunction and contributing to the sclerotic and fragile bone characteristics seen in PYCD [5].

The deficiency in osteoclast activity results in inadequate bone remodeling, thereby compromising bone integrity and increasing brittleness. The disorder is defined by characteristics such as low stature, osteosclerosis, micrognathia, open fontanelles, uneven teeth with hypodontia, a grooved palate, an obtuse mandibular angle, acroosteolysis of the distal phalanges resulting in shorter fingertips, and grooved nails [6].

Due to the infrequency of the disease, there is a deficiency of guidance about fracture therapy in PYCD. To date, only a limited number of case reports have been documented. Currently, there is no specific treatment for PYCD. Surgical interventions in these patients face challenges such as difficulty performing intramedullary nailing due to sclerosis of the medullary canal and stiffness encountered with locking plates [7]. Like our case, intramedullary nailing is a complex and time-consuming procedure that carries risks of complications. Careful surgical planning is essential to select the appropriate implant for each patient. Additionally, because the bone in these patients tends to be especially rigid, constant irrigation of the drill during surgery is critical to prevent temperature rise, which can cause bone necrosis and hinder healing. It is also important that the nail extends as proximally and distally as possible to minimize the high risk of refracture [8]. Perforating the sclerotic bone is exceedingly challenging. It requires a sufficient quantity of sharp and robust drill bits. Moreover, it is a labor-intensive task, and the complication of heat necrosis resulting from excessive drilling exacerbates the issue [2].

These recommendations align with a recent systematic review indicating that surgical treatments, including intramedullary nailing or plate fixation, should provide durable long-term support, although the overall evidence quality remains low [9]. In some instances, it may not be advised to remove hardware after bone healing, especially in femoral shaft fractures [10].

The increased skeletal fragility in this condition, along with the high incidence of fractures and refractures, may be attributed to an imbalance characterized by elevated bone formation coupled with decreased bone resorption. When refractures occur, treatment approaches largely rely on the surgeon’s expertise. These findings underline the importance of long-term monitoring in affected patients [11].

## Conclusions

Managing PYCD is particularly challenging due to the disorder’s complex impact on bone physiology. This case adds valuable insight into the limited surgical fracture treatment literature in PYCD by emphasizing internal plate fixation as a reliable and effective method for achieving stable fracture healing in these patients. Internal plate fixation provides the rigid stabilization necessary for overcoming the delayed healing process intrinsic to the disorder. Although technical challenges exist, such as difficulty in drilling dense sclerotic bone and managing the narrow medullary canal, it remains a preferred method because it offers durable fixation, reducing the risk of malunion and refractures compared to other fixation techniques.

Moreover, this case illustrates the importance of long-term follow-up in managing patients with PYCD undergoing fracture repair. Refractures can occur years after the initial surgery, necessitating continued surveillance and possibly additional interventions. Currently, no standardized treatment protocols exist, yet accumulating clinical evidence advocates for surgical approaches that provide sustained mechanical stability, such as internal plate fixation, as essential in optimizing outcomes.
